# Effects of the administration of Elovl5-dependent fatty acids on a spino-cerebellar ataxia 38 mouse model

**DOI:** 10.1186/s12993-022-00194-4

**Published:** 2022-08-06

**Authors:** Ilaria Balbo, Francesca Montarolo, Federica Genovese, Filippo Tempia, Eriola Hoxha

**Affiliations:** 1 Neuroscience Institute Cavalieri Ottolenghi (NICO), Regione Gonzole 10, 10043 Orbassano, Italy; 2 Department of Neuroscience, University of Torino, Torino, Italy; 3 Department of Molecular Biotechnology and Health Sciences, University of Torino, Torino, Italy

**Keywords:** Elovl5, Spino-cerebellar ataxia, SCA38, Motor deficits, Polyunsaturated fatty acids

## Abstract

**Background:**

Spinocerebellar ataxia 38 (SCA38) is a rare autosomal neurological disorder characterized by ataxia and cerebellar atrophy. SCA38 is caused by mutations of *ELOVL5* gene. *ELOVL5* gene encodes a protein, which elongates long chain polyunsaturated fatty acids (PUFAs). Knockout mice lacking *Elovl5* recapitulate SCA38 symptoms, including motor coordination impairment and disruption of cerebellar architecture. We asked whether, in Elovl5 knockout mice (*Elovl5*^*−/−*^), a diet with both ω3 and ω6 PUFAs downstream Elovl5 can prevent the development of SCA38 symptoms, and at which age such treatment is more effective. *Elovl5*^*−/−*^ mice were fed either with a diet without or containing PUFAs downstream the Elovl5 enzyme, starting at different ages. Motor behavior was assessed by the balance beam test and cerebellar structure by morphometric analysis.

**Results:**

The administration from birth of the diet containing PUFAs downstream Elovl5 led to a significant amelioration of the motor performance in the beam test of *Elovl5*^*−/−*^ mice, with a reduction of foot slip errors at 6 months from 2.2 ± 0.3 to 1.3 ± 0.2 and at 8 months from 3.1 ± 0.5 to 1.9 ± 0.3. On the contrary, administration at 1 month of age or later had no effect on the motor impairment. The cerebellar Purkinje cell layer and the white matter area of *Elovl5*^*−/ −*^mice were not rescued even by the administration of diet from birth, suggesting that the improvement of motor performance in the beam test was due to a functional recovery of the cerebellar circuitry.

**Conclusions:**

These results suggest that the dietary intervention in SCA38, whenever possible, should be started from birth or as early as possible.

## Introduction

*ELOVL5* (ELOngase of Very Long-chain fatty acids 5) encodes for an enzyme necessary to synthetize ω3 and ω6 very long polyunsaturated fatty acids (PUFAs), including arachidonic acid (ARA, C20:4, ω6) and docosahexaenoic acid (DHA, C22,6, ω3) [1]. Compelling evidence indicates an important role of PUFAs in brain signaling, ranging from membrane excitability to synaptic properties regulation. The brain contains high levels of PUFAs, that modulate membrane signaling dynamics, synaptogenesis [2, 3], synaptic transmission [4, 5], synaptic plasticity [6] and neuronal excitability [7, 8].

ELOVL5 belongs to a family of multi-pass transmembrane proteins (ELOVL1-7) linked with various nervous system diseases [9–20]. Missense mutations of *ELOVL5* cause the spinocerebellar ataxia type 38 (SCA38), a recently discovered rare form of ataxia characterized by ataxia, hyposmia, peripheral neuropathy and cerebellar atrophy [1, 21]. SCA38 is progressive and debilitating, as in three decades from the diagnosis, patients become unable to feed themselves and to walk [1].

*Elovl5 knockout (Elovl5*^*−/−*^ *)* mice recapitulate the main features of SCA38 [22]. Moreover, we recently demonstrated that *Elovl5* is highly expressed in the adult mice central nervous system and that, in the peripheral nervous system, its loss leads to dysfunctional action potential propagation and disrupted lipidic profile [23, 24]. More specifically, in patients with SCA38 and in *Elovl5*^*−/−*^ mice, both ω3 and ω6 PUFAs with more than 18 carbon atoms are reduced in all types of phospholipids, including phosphatidylcholines, phosphatidylethanolamines, phosphatidylserines, phosphatidylinositols, phosphatidic acids, sphingomyelins, ceramides, sulfatides, plasmalogens [24].

A few clinical trials on SCA38 patients have shown that the administration of DHA alone, which is the most abundant ω3 PUFA in the brain and can function as a precursor of other ω3 PUFAs, is sufficient to partially ameliorate ataxic symptoms [25, 26]. However, the effect of a more balanced and complete dietary supplementation, containing both ω3 and ω6 PUFAs downstream of Elovl5, is currently unknown. The aim of the present research was to test, in* Elovl5*^−/−^ mice, the extent of the beneficial effects, on motor performance in the balance beam test, of a diet with both ω3 and ω6 long chain PUFAs, which constitutes a complete supply of products downstream Elovl5.

## Results

### Progression of motor impairment of* Elovl5*^−/−^ mice fed with PUFA precursors only diet

**Fig. 1:**
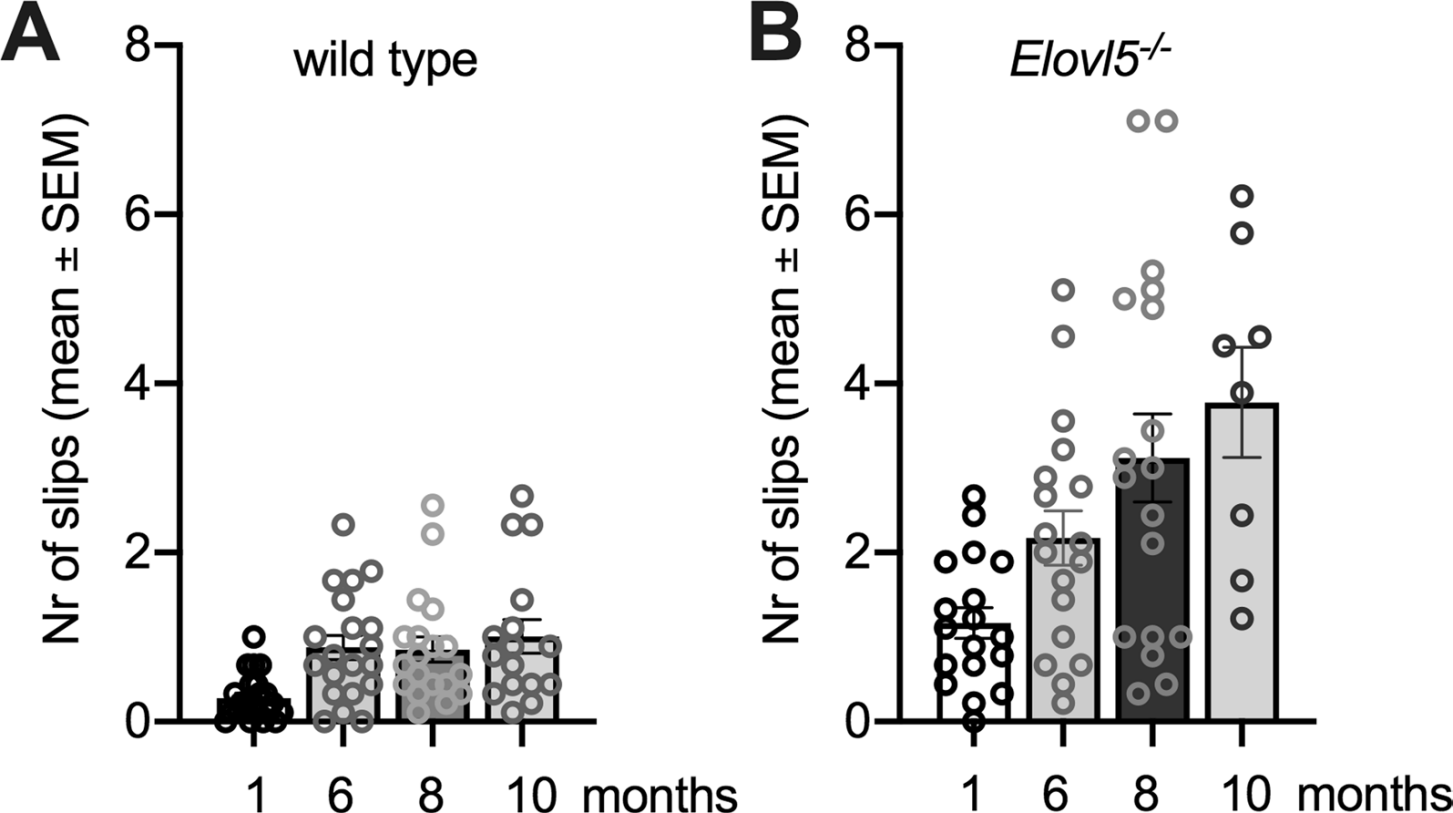
Progression of motor impairment in *Elovl5*^−/−^ mice administered with a PUFA precursors diet. Bar graphs showing the number of errors at different ages (from 1 to 10th month of age) for wild type and *Elovl5*^*−/−*^ mice. *Elovl5*^*−/−*^ mice undergo a great worsening of motor coordination across months, while wild type mice, after the first month of age, show an almost unchanged mean number of errors with age (****P < 0.0001, Two-way ANOVA; n = 23 wild type mice, n = 18 *Elovl5*^*−/−*^ mice). Values are mean ± S.E.M

A deficit in the balance beam test was already present at the age of 1 month for *Elovl5*^*−/−*^ mice, as shown by a higher number of paw placement errors compared to wild type littermates (Fig. 1). The difference of motor performance in such test between genotypes remained statistically significant throughout the ages analyzed (P < 0.0001, Two-way ANOVA; n = 38 wild type mice: 0.24 ± 0.04; n = 38 *Elovl5*^*−/−*^ mice: 0.86 ± 0.11) (Fig. 1). *Elovl5*^*−/−*^ mice showed a progressive increase of foot slip errors with age, in agreement with the worsening of *Elovl5*^*−/−*^ mice motor performance reported by Hoxha et al. [22].

### Effects of the administration of a complete PUFA diet since birth on *Elovl5*^−/−^

Wild type and *Elovl5*^*−/−*^ mice born from dams that received a complete PUFA diet, also containing long chain PUFAs, were evaluated on the balance beam test.

**Fig. 2:**
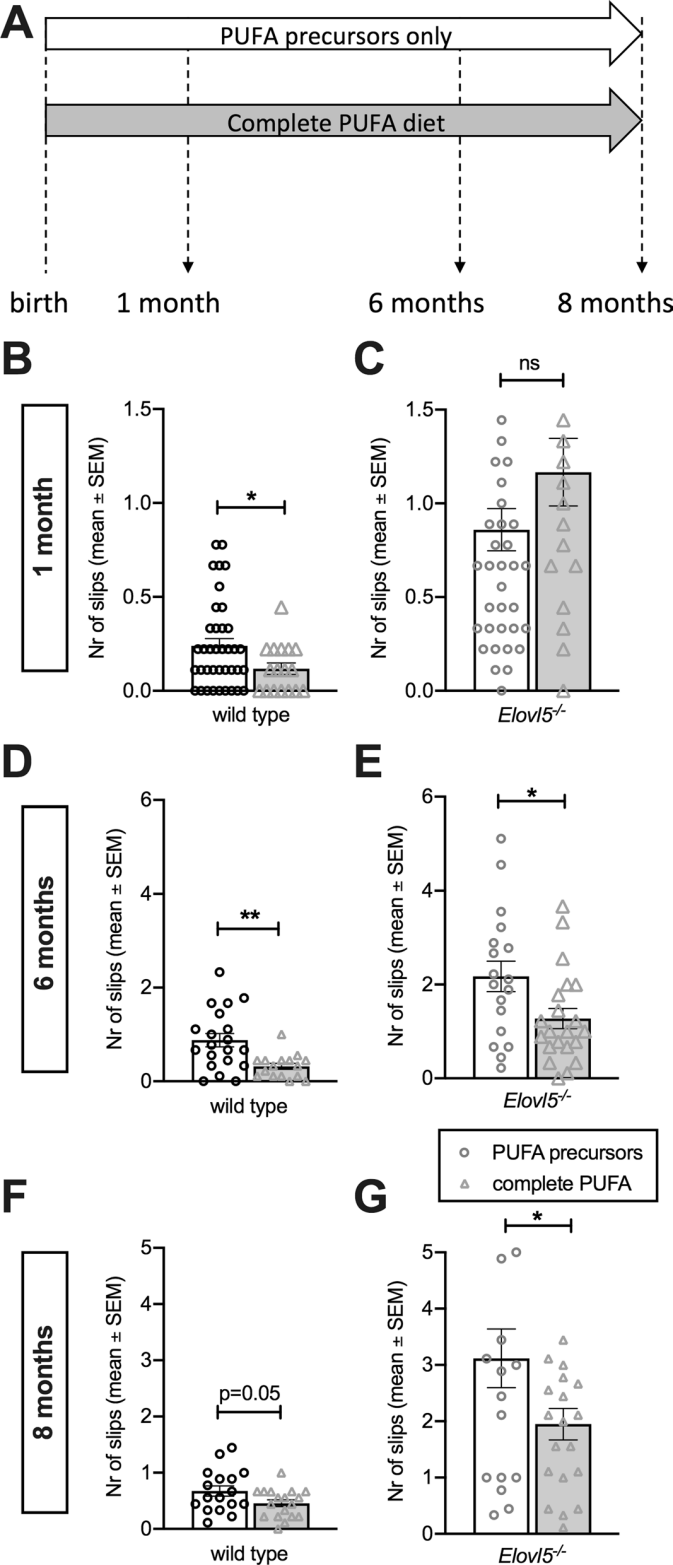
Administration of a complete PUFA diet since birth on Elovl5 ^−/−^ mice slows down motor impairment.** A** Scheme of diet administration: one group composed of *Elovl5*^*−/−*^ (n = 18) and wild type mice (n = 17) received a diet providing them with both Elovl5 substrates and downstream products from birth (complete PUFA diet), while the other group of *Elovl5*^*−/−*^ (n = 38) and wild type mice (n = 38) received only PUFA precursors from birth (PUFA precursors only). **B**, **C** Bar graphs showing errors committed traversing the experimental apparatus at 1 month of age for wild type and *Elovl5*^*−/−*^mice who received the two different diets (for wild type mice: *P < 0.05; for *Elovl5*^*−/−*^mice P > 0.05, Unpaired Student’s t-test). **D**, **E** Motor performances at 6 months of age for *Elovl5*^*−/−*^mice and wild type littermates receiving the two different diets from birth (for wild type mice: **P < 0.01; for *Elovl5*^*−/−*^mice *P < 0.05, Unpaired Student’s t-test). **F**, **G** Motor performances at 8 months of age for *Elovl5*^*−/−*^ mice and control littermates compared to genotype matched PUFA precursors mice (P < 0.05, Unpaired Student’s t-test). Values are mean ± S.E.M

In wild type mice, the administration of a complete PUFA diet reduced the number of foot slips at 1 (P < 0.05, Unpaired Student’s t-test t_(53)_ = 2.02; Fig. 2A and B) and 6 months (P < 0.01, Unpaired Student’s t-test t_(34)_ = 3.29; Fig. 2D), while at 8 months there was no significant difference (P = 0.05, Unpaired Student’s t-test; Fig. 2F). With 2-way repeated measures ANOVA analysis, there was no significant effect of diet (P > 0.05; F(1,46) = 0.96) while the effect of time was significant [P < 0.001; F=(1.61, 60.25) = 10.71].

In *Elovl5*^*−/−*^ mice, at 1 month the complete PUFA diet did not significantly change the number of foot slips (P > 0.05, Unpaired Student’s t-test; Fig. 2C). However, at 6 and 8 months, *Elovl5*^*−/−*^ mice with a complete PUFA diet showed a significantly better performance at the beam test compared to *Elovl5*^*−/−*^ mice kept with PUFA precursors only (for 6 months old mice: P < 0.05, Unpaired Student’s t-test t_(37)_ = 2.37, Fig. 2E; for 8 months old mice: P < 0.05, Unpaired Student’s t-test t_(36)_ = 2.03). With 2-way repeated measures ANOVA analysis, there a significant effect of both diet (P < 0.01; F(1,41) = 10.72) and time (P < 0.0001; F=(1.73, 59.51) = 14.13).

### Effects of the administration of a complete PUFA diet at later ages on* Elovl5*^−/−^ mice

**Fig. 3:**
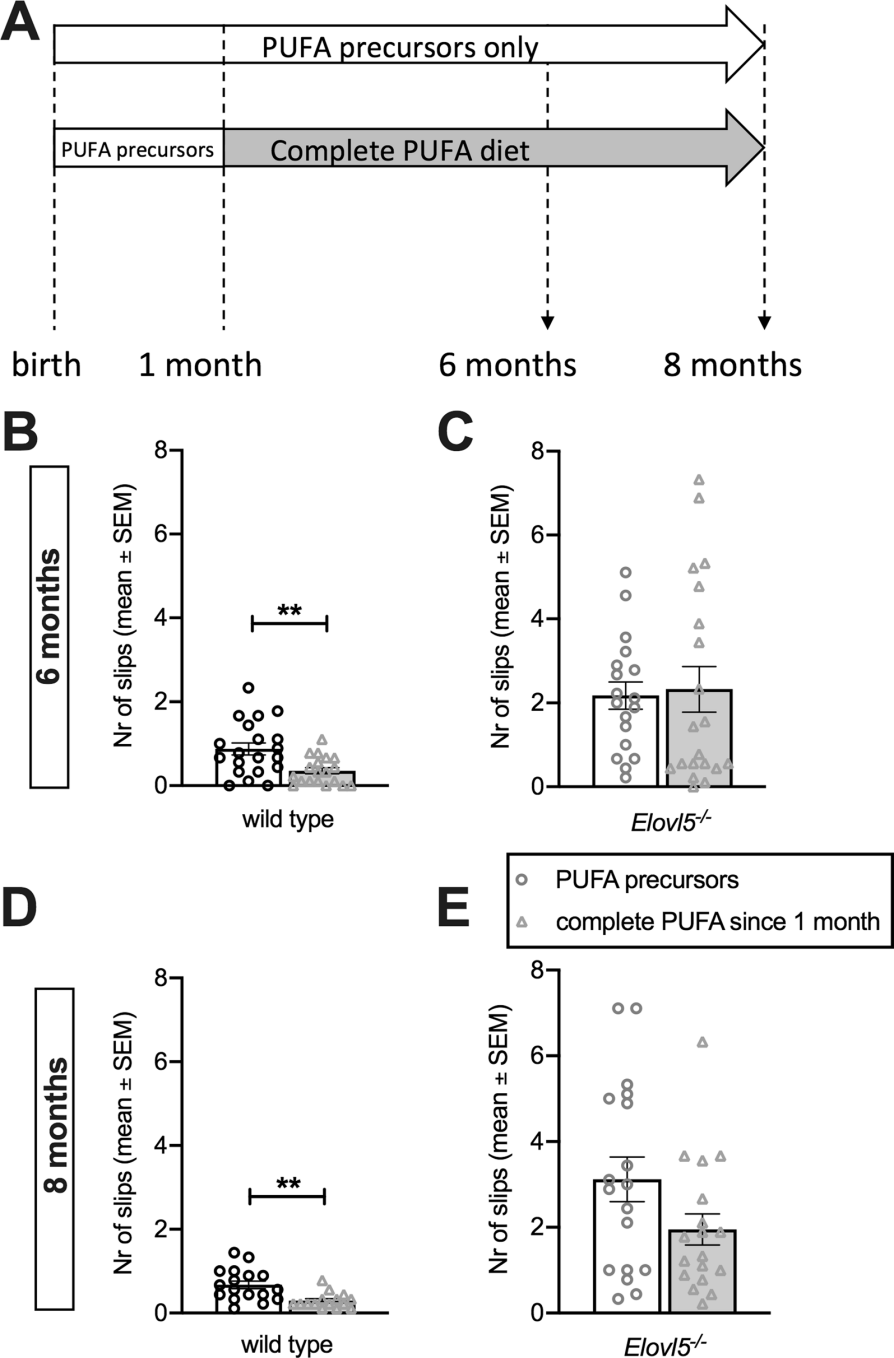
Administration of a complete PUFA diet from 1 month of age in *Elovl5*^*−/−*^ and wild type mice. **A** Scheme of diet administration: one group composed of *Elovl5*^*−/−*^ (n = 20) and wild type mice (n = 19) received a diet providing them with both Elovl5 substrates and downstream products from 1 month of age (complete PUFA diet), while the other group of *Elovl5*^*−/−*^ (n = 18) and wild type mice (n = 20) received PUFA precursors only from birth (PUFA precursors only). **B**, **C** Bar graphs showing errors committed at 6 months of age when traversing the experimental apparatus for wild type and *Elovl5*^*−/−*^mice who received the complete diet since 1 month of age compared to the group receiving the precursor diet only (for wild type mice: **P < 0.01, Unpaired Student’s t-test; for *Elovl5*^*−/−*^mice P > 0.05, Unpaired Student’s t-test). **D**, **E** Bar graphs showing errors committed at 8 months of age when traversing the experimental apparatus by wild type and *Elovl5*^*−/−*^ mice receiving the two different diets (for wild type mice: **P < 0.01, Unpaired Student’s t-test; for *Elovl5*^*−/−*^mice P = 0.07, Unpaired Student’s t-test). Values are mean ± S.E.M

The administration to wild type mice of a complete PUFA diet starting at 1 month of age significantly reduced the number of foot slips compared to wild type mice fed with PUFA precursors only (for 6 months old mice: P < 0.01, Unpaired Student’s t-test t_(37)_ = 3.18, Fig. 3A and B; for 8 months old mice: P < 0.01, Unpaired Student’s t-test t_(30)_ = 3.52), Fig. 3D). In contrast, the complete PUFA diet starting at 1 month of age, failed to improve the beam test motor performance of *Elovl5*^−/−^ mice (P > 0.05, Unpaired Student’s t-test; Fig. 3C and E), although at 8 months there was a trend to perform fewer errors than *Elovl5*^−/−^ mice with PUFA precursors only (Fig. 3E). However, it should be noticed that PUFA precursors *Elovl5*^−/−^ mice have a tendency to worsen their performance from 6 to 8 months, while, in the same period, *Elovl5*^−/−^ mice with complete PUFAs since 1 month retained the same performance level, without worsening.

**Fig. 4:**
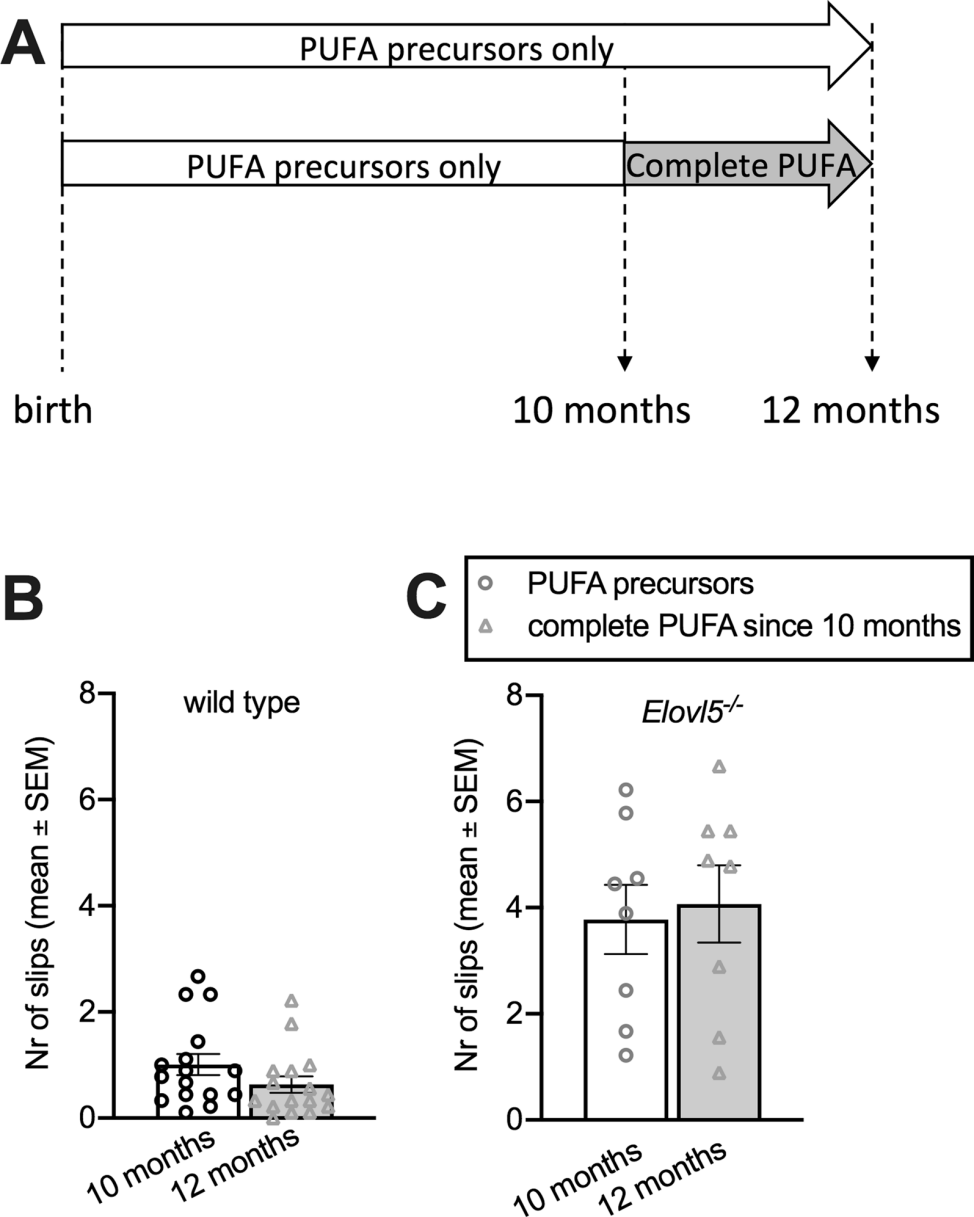
Administration of a complete PUFA diet on *Elovl5*^*−/− *^mice in late adulthood does not ameliorate motor deficits.** A** Schematic representation of the experimental design. One group composed of 10 months old *Elovl5*^*−/−*^ (n = 8) and wild type mice (n = 16) received the complete PUFA diet since 10 months of age and the motor performance was checked after two months of administration. **B**, **C** Mean number of errors committed traversing the experimental apparatus for both wild type and *Elovl5*^*−/−*^ mice at 10 (white bars) and 12 months (grey bars). As shown, the complete diet administered in late adulthood fails to rescue motor coordination impairment of *Elovl5*^*−/−*^ mice (for both wild type and *Elovl5*^*−/−*^ mice: P > 0.05, Unpaired Student’s t-test). Values are mean ± S.E.M

In order to understand whether a later start of the complete PUFA diet could provide a benefit, similar to a therapy started after diagnosis of SCA38 in an adult individual, in a separate group we administered the complete PUFA diet since 10 months of age and continued for two months. With this protocol, neither wild type nor *Elovl5*^*−/−*^ mice showed an improvement relative to the respective controls receiving PUFA precursors only (P > 0.05, Unpaired Student’s t-test; Fig. 4A, B and C).

### Effects of the complete PUFA diet on* Elovl5*^−/−^ cerebellar morphological parameters

In a previous report [22] we showed that *Elovl5*^*−/−*^ cerebellar cortex has a shorter length of the PC layer and a reduced section area of the cerebellar white matter. Therefore, in 12 months old mice, we studied the effects of the complete PUFA diet administered either since birth or since 1 month of age on these morphological parameters. The complete PUFA diet failed to affect these parameters in either wild type or *Elovl5*^*−/−*^ mice. Thus, in *Elovl5*^*−/−*^ the complete PUFA diet, even since birth, failed to increase either the PC layer length (*Elovl5*^*−/−*^ mice: PUFA precursors diet, n = 8 mice, 20.11 ± 0.55 mm; complete PUFAs since birth, n = 8 mice, 19.86 ± 0.30 mm, complete PUFAs since 1 month: n = 11 mice, 20.17 ± 0.22 mm; P > 0.05 for all comparisons, Unpaired Student’s t-test) or the white matter area (*Elovl5*^*−/−*^ mice: PUFA precursors diet, n = 8 mice, 0.12 ± 0.003 mm^2^; complete PUFAs since birth, n = 8 mice, 0.12 ± 0.003 mm^2^; complete PUFAs since 1 month, n = 11 mice, 0.11 ± 0.004 mm^2^; P > 0.05 for all comparisons, Unpaired Student’s t-test).

## Discussion

In this work, we assessed the effects of a complete PUFA diet, containing both ω3 and ω6 downstream products of Elovl5, on motor performance and on cerebellar architecture in SCA38 murine model (*Elovl5-/-* mice). We show that the introduction of the complete PUFA diet at birth is more effective than a later administration in ameliorating the motor performance in the beam test of *Elovl5-/-* mice. The improvement of the motor performance is not paralleled by changes of cerebellar PC layer length and white matter area.

Most hereditary ataxias still lack a specific cure able to revert the progressive worsening of motor functions. Because of this almost complete lack of clinically relevant treatments, several studies have been conducted in animal models of specific types of SCA [27]. In SCA1 Hourez et al. [28] found increased A-type voltage-dependent potassium current, which could be blocked by aminopyridines, improving the motor performance, and partially protecting against cell atrophy. Notartomaso et al. [29] showed that, in a SCA1 model mouse, the metabotropic glutamate receptor 1 (mGlu1) receptor is reduced in the cerebellum, and that ataxic symptoms could be reverted by administration of a mGlu1 receptor positive allosteric modulator. On the same line, Shuvaev et al. [30] administered baclofen to enhance mGlu1 receptor function and this treatment improved the motor performance of SCA1 model mice. In contrast, in SCA2 model mice, the mGlu1 receptor signaling pathway is enhanced, leading to Purkinje cell degeneration [31]. Inhibition of the mGlu1 receptor-signaling pathway by dantrolene restored the normal functional level of this pathway and improved motor symptoms [31].

Recently, a few attempts of pharmacological treatments have been performed in patients of mixed groups of dominant and recessive ataxias, showing some weak and short-lasting benefit with riluzole and thyrotropin releasing hormone [32]. In patients with SCA3, valproic acid showed some effect, which needs to be further evaluated [33]. Clinical trials utilizing repetitive transcranial cerebellar magnetic stimulation (rTMS) or anodal transcranial direct current stimulation (tDCS) showed improvements of symptoms in patients with different types of ataxia, but more studies are needed before these protocols can be broadly applied in clinical settings [34]. Of great impact in the ataxia field research is the clinical trial performed in symptomatic SCA38 patients where the diet supplementation of DHA, one of the PUFAs downstream Elovl5, was sufficient to ameliorate and stabilize clinical symptoms [25, 26].

There is a limited number of chronic progressive ataxias that, like SCA38, can be ameliorated by dietary administration of compounds which are deficient in patients [35]. Like SCA38, in which a loss of function of an enzyme brings about a reduction of downstream products [24], ataxia with vitamin E deficiency (AVED) is caused by mutation of alpha tocopherol transfer protein gene [36], with the consequence that vitamin E cannot be incorporated in very low density apolipoproteins [37]. AVED can be treated by Vitamin E supplementation [38, 39]. Such therapy is currently administered to patients with AVED and proved to be effective in slowing down or stopping the progression of ataxic symptoms in the majority of patients. A similar approach was used also in Cerebrotendinous xanthomatosis (CTX), a lipid storage disorder, caused by mutations of the mitochondrial sterol 27-hydroxylase enzyme [40]. The CTX patients have a reduced amount of hepatic-bile acids (cholic and chenodeoxycholic acid). The simple oral supplementation of chenodeoxycholic acid can ameliorate the symptoms of CTX patients [41]. Another type of treatable disease characterized by ataxia is Abetalipoproteinemia, which is caused by the mutation of the microsomal triglyceride transfer protein (MTTP). The mutation of MTTP leads to low levels of plasma apolipoprotein B containing lipoproteins, causing a poor absorption of fat-soluble vitamins like vitamin E and A. Treatment with vitamin E and A, and a low-fat diet are reported to slow the neurological signs in this disease [42].

The motor deficit of *Elovl5*^*−/−*^ mice was significantly improved by the introduction of the downstream products of Elovl5 in early phases of the disease suggesting being more beneficial in pre-symptomatic stage. This earlier intervention was able to significantly slow down the progression of ataxia. In a similar way as reported here, early administration of the treatment in young AVED, CTX and Abetalipoproteinemia patients, was more effective in improving neurological symptoms [35, 39, 41]. These clinical trials have highlighted the mandatory necessity to start the treatment as early as possible to obtain the amelioration of the neurological symptoms.

Our results show that the complete PUFA diet has also a positive impact on the motor performance of wild type mice, suggesting that the effect of PUFA is not restricted to pathological conditions. Indeed, it is widely known that the supplementation of PUFAs is beneficial for the development and function of the central nervous system [42].

The motor improvement induced by the complete diet in *Elovl5-/-* mice is not paralleled by an amelioration of the cerebellar atrophy, as measured by PC layer length and white matter area. This result suggests that the complete diet induces a functional recovery of cerebellar circuitry while a morphological amelioration is not present for the parameters measured. This is consistent with the increased cerebellar metabolism observed in SCA38 patients supplemented with DHA [25, 26]. However, further experiments are needed to support this hypothesis.

## Conclusions

In conclusion, our results suggest that the intervention with a complete diet containing both ω3 and ω6 Elovl5 downstream products, when administered in the initial phase of SCA38 is the most effective approach to slow down the progression of the disease and highlights the need for an earlier diagnosis of SCA38. Moreover, for female patients diagnosed with SCA38, is it advisable to start diet supplementation with ELOVL5 downstream PUFAs already during pregnancy.

## Materials and methods

### Animals

All experimental procedures have been authorized by the Italian Ministry of Health (authorization number: 161/2016-PR). Elovl5 knockout (*Elovl5*^*−/−*^) mice have been kindly provided by Dr. Moon and Dr. Horton of the UT Southwestern Medical Center and bred in our Animal Facility at NICO. *Elovl5*^*−/−*^ and wild type mice (C57Bl6 background) were used for all the experimental paradigms, while heterozygous mice were only used as breeders. Data from female and male mice were pooled together because they showed no significant difference.

### Mice experimental groups

To assess the effect of a PUFA rich diet in our pathological model we divided mice in three groups based on the different time for diet administered.

The first group, “PUFA precursors diet”, was composed of mice born from dams kept on a natural diet without animal derivatives, containing essential PUFAs like linoleic and α-linolenic acids, but excluding the presence of ω3 and ω6 PUFAs downstream Elovl5, such as DHA, eicosapentaenoic acid (EPA) and ARA (Teklad Global 18% Protein Rodent Diet, Harlan Laboratories) (Table 1). The dams received the natural diet from the moment of mating and throughout pregnancy and lactation. The offspring continued to be fed throughout life with the “PUFA precursors diet”.

**Table 1:** Fatty acids content of PUFA only and complete diets

Fatty acid	PUFA precursors diet (Teklad Global 18% Protein Rodent Diet, Harlan Laboratories)	Complete PUFA diet (4RF25, Mucedola Srl)
Linoleic acid C18:2n-6	2227.234 ± 48.492	1542.747 ± 183.529
Gamma-Linolenic acid C18:3n-6	325.807 ± 11.766	302.807 ± 25.603
Arachidonic acid C20:4n-6	0.254 ± 0.031	9.901 ± 0.365
Eicosapentaenoic acid C20:5n-3	0.190 ± 0.034	28.677 ± 1.564
Docosahexaenoic acid C22:6n-3	0.096 ± 0.027	125.449 ± 1.686
Oleic acid (C18:1)	1920.966 ± 68.373	1847.310 ± 174.768
Erucic acid (C22:1)	15.155 ± 0.361	12.909 ± 0.392
Nervonic acid (C24:1)	2.081 ± 0.170	2.203 ± 0.024
Palmitic acid (C16:0)	1243.140 ± 50.405	875.832 ± 70.505
Margaric acid (C17:0)	10.233 ± 0.180	11.495 ± 0.606
Stearic acid (C18:0)	256.665 ± 4.021	241.530 ± 11.131
Arachidic acid (C20:0)	17.404 ± 0.034	18.555 ± 1.564
Behenic acid (C22:0)	16.268 ± 0.732	14.383 ± 0.236

Data are expressed as ng/mg (mean ± standard error)

The second group of mice was born from dams receiving a complete PUFA diet, containing precursors and downstream products of Elovl5, (Standard rodent diet 4RF25, Mucedola Srl) (Table 1), from the moment of mating and throughout pregnancy and lactation. The offspring continued to be fed throughout life with the complete diet.

The third group of mice was born from dams receiving the natural diet with PUFA precursors only from the moment of mating and throughout pregnancy and lactation. The offspring was divided in two subgroups and were switched to a complete PUFA diet at different ages. One subgroup was switched at 1 month, while another subgroup at 10 months of age. Each group included both *Elovl5*^*−/−*^ and wild type littermates, equally representing both sexes.

### Balance beam test

To check the effect of complete PUFA diet on motor performance we performed balance beam test at 1, 6, 8 months of age in *Elovl5*^*−/−*^ and wild type mice. The beam test was performed as previously described [44]. Briefly, we used a metal beam 1 cm wide, and 100 cm long suspended 12 cm above the bench. The mice had to cross the beam to reach a cage enriched with toys. To allow the mice to familiarize with the experimental apparatus, thus reducing the stress due to an unknown procedure, the experiment was preceded by 3 days of acclimation. On the day of the test, the animals were placed to acclimate in the behavioral room at least 15 min before the experiment. Both *Elovl5*^*−/−*^ and wild type mice were tested individually, and each animal was encouraged to traverse the beam at least three times. The test was repeated for three consecutive days. The test was recorded using a video camera and analyzed offline by an operator blind to the genotype. The number of slips from the beam was measured to assess the motor performance.

### Histological procedures

We analyzed the cerebellar cortex of *Elovl5*^*−/−*^ and wild type littermates fed with the three different diets at 12 months of age. Histological procedures were performed as previously described [45]. Animals were anesthetized using a cocktail of ketamine (100 mg/kg body weight) and xylazine (10 mg/kg body weight) via intraperitoneal injection. The mice were intracardially perfused initially with a physiological solution (NaCl 0.9%) and then with 4% paraformaldehyde in 0.12 M phosphate buffer, pH 7.2–7.4. Following perfusion, the brains were removed and stored at 4 °C for 24 h immersed in the same fixative. The brains were then transferred to a cryoprotectant solution made of 30% sucrose in 0.12 M phosphate buffer for few days. For each mouse, the cerebellum was separated and embedded in optimal cutting temperature compound, frozen in ice-cold isopentane. Cerebella were serially cut by a cryostat in 30 μm-thick sagittal slices and collected in phosphate buffered saline (PBS).

Cresyl Violet Staining (Nissl Staining) was performed on one series for each *Elovl5*^*−/−*^ and wild type mouse at 12 months of age. Free-floating sections were washed twice in PBS (15 min each). The series were mounted on gelatin-coated slides and let air dry overnight. The staining was performed as follows: mounted series were washed for 2 min in distilled water to remove any residual salts and then stained in 0.1% Cresyl violet solution for 15 min. Following, sections were rinsed again in distilled water for 2 min, and then dehydrated using a series of alcohols: 50% (2 min), 70% (2 min), 95% (I) (2 min), 95% (II) (few seconds) and 100% (2 min). Next, the gelatin-coated slides were immersed in xylene for 5 min and finally a clear glass coverslip was applied using a permanent mounting medium. We evaluated for each slice white matter area/total area of the slice ratio and the PC layer length. At least three vermal slices/animal and three animals were analyzed. All measurements were done blind relative to the mouse genotype.

### Statistical analysis

Statistical analyses were carried out with GraphPad Prism 9 software (GraphPad Software Inc., San Diego, CA, USA). Data were tested for normal distribution with Kolmogorov-Smirnov test. When data were normally distributed two-tailed unpaired Student’s t-test followed by Holm-Sidak post hoc correction was used. For data not normally distributed, Mann-Whitney U test was used. Two-way ANOVA followed by the Bonferroni test was used when indicated. All data are expressed as mean ± SEM. P < 0.05 was considered as statistically significant.

## Data Availability

The data are available for any scientific use from the corresponding author on reasonable request.

## Abbreviations

SCA38: : Spinocerebellar ataxia 38
ELOVL5: ELOngase of Very Long-chain fatty acids 5
PUFAs: very long polyunsaturated fatty acids
ARA: arachidonic acid
DHA: docosahexaenoic acid
ELOVL5^-/-^: * Elovl5 knockout *
rTMS: transcranial cerebellar magnetic stimulation
tDCS: transcranial direct current stimulation
AVED: ataxia with vitamin E deficiency
CTX: Cerebrotendinous xanthomatosis
MTTP: microsomal triglyceride transfer protein
PBS: phosphate buffered saline
mGlu1: metabotropic glutamate receptor 1
